# Novel positive allosteric modulators of alpha 5 subunit-containing GABA_A_ receptors (α5-GABA_A_Rs) reverse the hyperdopaminergic state in a neurodevelopmental model of schizophrenia

**DOI:** 10.1016/j.schres.2026.02.010

**Published:** 2026-02-17

**Authors:** Daniela L. Uliana, Mariana O. Popa, Michael Paradowski, Karen T. Elvers, Marcus Hanley, Alex Baldwin, John R. Atack, Anthony A. Grace

**Affiliations:** a Departments of Neuroscience, Psychiatry and Psychology, University of Pittsburgh, Pittsburgh, PA, USA; b Medicines Discovery Institute, Cardiff University, Park Place, CF10 3AT, Cardiff, Wales, United Kingdom; c Draig Therapeutics, Sbarc Building, Maindy Road, CF24 4HQ, Cardiff, Wales, United Kingdom

**Keywords:** Dopamine, Schizophrenia, α5-GABAAR, Positive allosteric modulators

## Abstract

Dysfunction in the GABAergic system has been described in schizophrenia, including decreased expression of α5 subunit-containing GABA_A_ receptors (α5-GABA_A_Rs) in patients with schizophrenia. This study explores the therapeutic potential of positive allosteric modulators (PAMs) of the α5-GABA_A_R to reduce the hyperdopaminergic state produced by the neurodevelopmental methylazoxymethanol acetate (MAM) model of schizophrenia. Male offspring rats generated from pregnant females injected with saline or MAM at gestational day 17 were used for the electrophysiological recordings as adults. *In vivo* electrophysiological recordings were performed to assess the effects of 10 mg/kg of the novel α5-GABA_A_R-preferring PAM alogabat on dopamine (DA) neuron activity in the ventral tegmental area (VTA); a dose shown to produce sustained, ≥80% α5-GABA_A_R occupancy over a time period of 0.5–3.5 h post-dose. A less extensive confirmatory study was also performed with a second α5-GABA_A_R PAM, Compound 100. The primary outcome was that at a dose of 10 mg/kg, which corresponded to an α5-GABA_A_R occupancy of ≥80% for alogabat and 70% for Compound 100, reversed the increased number of spontaneously active DA neurons in MAM rats. Alogabat data showed that these effects were driven by a reduction in the central and lateral (but not medial) portions of the VTA; regions that project to the associative striatum. These findings suggest that selective targeting of α5-GABA_A_Rs may help normalize aberrant DA activity. The study highlights α5-GABA_A_Rs as a promising therapeutic target, potentially addressing positive symptoms by restoring excitatory-inhibitory balance in a key region of the brain implicated in the pathophysiology of schizophrenia.

## 1. Introduction

Dysfunction in the γ-Aminobutyric acid (GABA) system has been implicated in many psychiatric conditions, including schizophrenia (Bast et al., 2017; Chiapponi et al., 2016; Zhang et al., 2021). The GABA_A_ receptor is comprised of different subunit classes (α1–6, β1–3, γ1–3, δ, π, θ, and ε), with most receptors being comprised of α:β:γ subunits (Jacob, 2019; McKernan and Whiting, 1996; Möhler, 2006). GABA_A_Rs with a combination of α1-, α2-, α3- and α5βγ2 subunits contain an allosteric modulatory (benzodiazepine binding) site through which receptor function can be altered in a bidirectional (positive- and negative allosteric modulatory) manner.

Expression of the α5 subunit of the GABA_A_R family is enriched within the ventral hippocampus and amygdala with lower levels being expressed in prefrontal and thalamic areas (Fritschy and Panzanelli, 2014; Heldt and Ressler, 2007; Ramos et al., 2004; Serwanski et al., 2006). α5 subunit-containing GABA_A_Rs (α5-GABA_A_Rs) are mainly located on pyramidal neurons and regulate their baseline firing activity, being critical for excitatory-inhibitory balance (Semyanov et al., 2004). GABAergic neurotransmission and more particularly α5-GABA_A_Rs have been implicated in the pathogenesis of schizophrenia. For example, a decreased α5-GABA_A_R binding in the hippocampus is reported in unmedicated schizophrenia patients using *in vivo* PET imaging (Marques et al., 2021). Furthermore, in *post-mortem* brains of patients with schizophrenia, there are lower levels of GABA synthesizing enzyme GAD67 (Benes et al., 2007) and decreased numbers of parvalbumin-expressing interneurons in the hippocampus (Santos-Silva et al., 2023; Zhang and Reynolds, 2002). Finally, animal studies showed that lower α5-GABA_A_R function in the ventral hippocampus impacts behavioral response and dysregulate the neurobiological circuit in the disease state (Lieberman et al., 2018; Schobel et al., 2013; Sonnenschein et al., 2020), as the hippocampus is proposed to be the pathological site of schizophrenia (Uliana et al., 2023). Indeed, the ventral hippocampus regulates dopamine (DA) system activity within the ventral tegmental area (VTA) (Lodge and Grace, 2006); an area in which increased DA activity is observed and has been associated with positive symptoms of schizophrenia (Kätzel et al., 2020; Lieberman et al., 2018; Lodge and Grace, 2008, 2007).

**Fig. 1.**
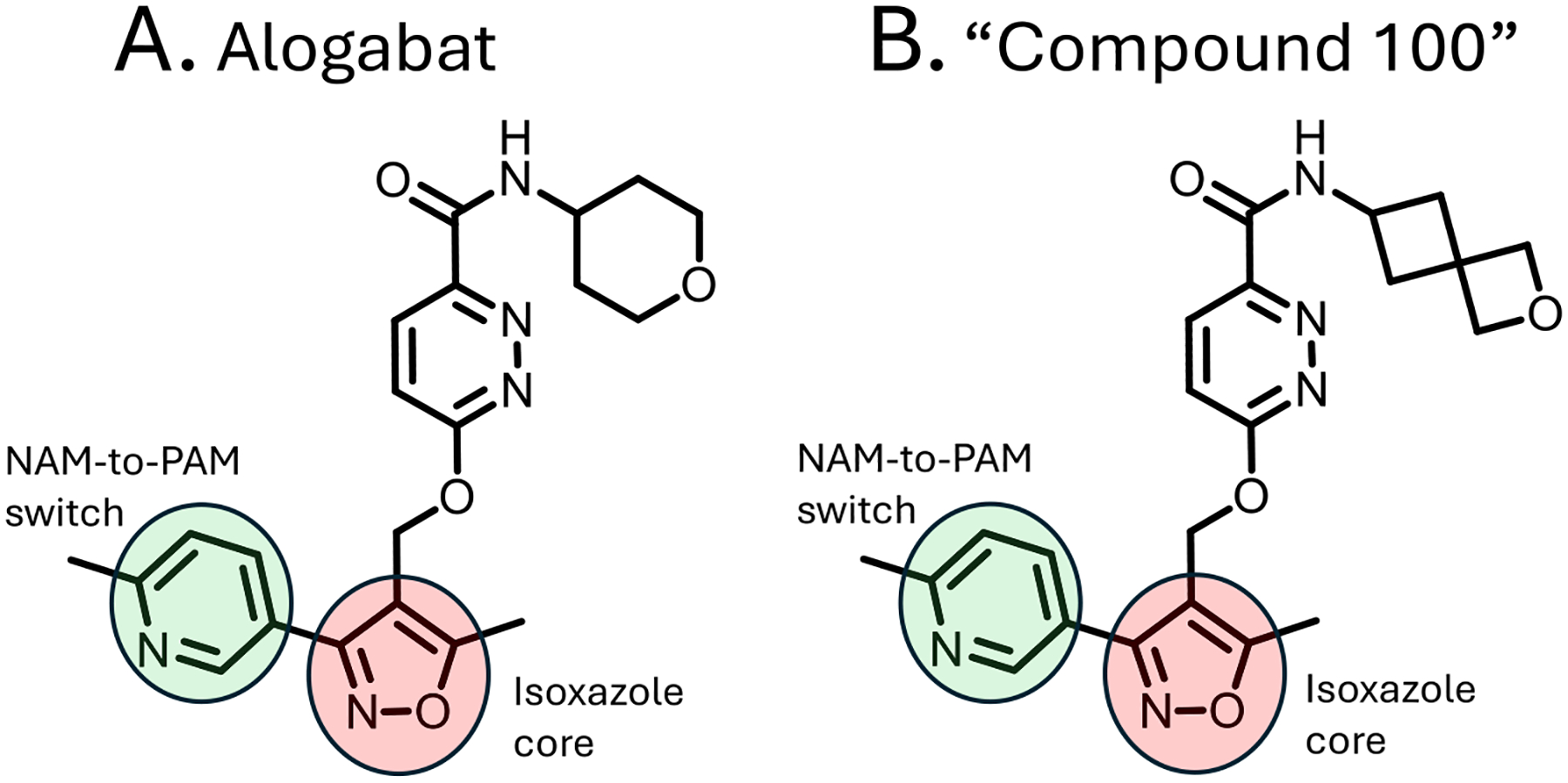
Structures of α5-GABA_A_R PAMs used in the present study. A. Alogabat, also described as RG7816 and RO7017773, (6-((5-methyl-3-(6-methylpyridin-3-yl) isoxazol-4-yl)methoxy)-*N*-(tetrahydropyran-4-yl)pyridazine-3-carboxamide). B. Compound 100 (6-((5-methyl-3-(6-methylpyridin-3-yl)isoxazol-4-yl)methoxy)-*N*-(2-oxaspiro[3.3]heptan-6-yl)pyridazine-3-carboxamide). Both compounds were identified from patent WO 2018/104419 A1 “New isoxazolyl ether derivatives as GABA A Alpha5 PAM” in which Alogabat is described as Compound 8. The isoxazole core is highlighted in pale red and the introduction of a pyridyl group, highlighted in green, confers the PAM-to-NAM switch in efficacy relative to the phenyl group found in α5-GABA_A_R NAMs such as Basmisanil (Hipp et al., 2021; Kasaragod et al., 2023).

Animal models have attempted to reproduce behavioral and neurobiological features of schizophrenia (Uliana et al., 2023, 2022). One model that relies on the neurodevelopmental origins of schizophrenia is the methylazoxymethanol acetate (MAM) model (Modinos et al., 2015). MAM is a DNA-methylating agent that is administrated to pregnant females on gestational day 17 (Moore et al., 2006). MAM offspring have several dysfunctions that could be translated to schizophrenia, such as increased sensitivity to psychostimulants, reduced prepulse inhibition of startle, and increased number of spontaneous active DA neurons in the VTA (Du and Grace, 2016; Lodge and Grace, 2008; Moore et al., 2006). In MAM rats hyperexcitability of the ventral hippocampus is observed, (Lodge and Grace, 2007) potentially due to a loss of GABAergic interneurons controlling pyramidal neuron activity (Gill and Grace, 2014). In addition, MAM animals have a decreased density of α5-GABA_A_Rs in the ventral hippocampus (Kiemes et al., 2022). Furthermore, positive allosteric modulators (PAMs) of this GABA_A_R subtype can normalize the increased DA activity in the VTA (Gill et al., 2011; Perez et al., 2023) and hyperlocomotion induced by amphetamine in MAM rats (Gill et al., 2011). Thus, the α5-GABA_A_R represents a critical GABA_A_R subtype that regulates different domains of the MAM phenotype such as dopamine-system hyperactivity (Donegan et al., 2019; Gill et al., 2011). Consistently, a genetic point mutation resulting in reduced expression of the α5-GABA_A_R subunit in the hippocampus or its antagonism is associated with behavioral dysregulation typically related to schizophrenia, such as altered prepulse inhibition to startle (Hauser et al., 2005) and disrupted latent inhibition (Gerdjikov et al., 2008). These data all suggest that enhancing the function of α5-GABA_A_Rs represents a novel strategy for the development of new treatments for schizophrenia. The aim of the present study was to test two new α5-GABA_A_R preferring PAMs in the hyperdopaminergic state observed in the MAM neurodevelopmental model. The two compounds used in the present study were reported by Roche in patent WO 2018/104419 as Example 8 (alogabat) and Example 100 (Compound 100; see Fig. 1 for structures) with alogabat being the primary compound with which to establish a Proof-of-Concept and Compound 100 being used as a second compound to further evaluate our hypothesis. The *in vitro* and *in vivo* (α5-GABA_A_R occupancy) properties of these compounds were also characterized. During the course of these studies, a separate publication describing the characterization of alogabat has been published (Cecere et al., 2025), which largely concur with part of our data. The data generated in the present study show that two different α5-GABA_A_R PAMs can attenuate the hyperdopaminergic state generated using the MAM model and further support the α5-GABA_A_R PAM mechanism as a novel approach to the treatment of the hyperdopaminergic state associated with schizophrenia.

## 2. Materials and methods

### 2.1. Compounds

Compounds were synthesized in house (Medicines Discovery Institute, Cardiff University), as described in more detail elsewhere (see Patent WO 2018/104419).

### 2.2. In vitro characterization of alogabat and Compound 100

#### 2.2.1. Radioligand binding

The binding affinities (Ki values) of alogabat and Compound 100 were measured at human α1β3γ2, α2β3γ2, α3β3γ2, α5β3γ2 GABA_A_Rs stably expressed in mouse fibroblast L cells using a [^3^H]Ro15–1788 ([^3^H] flumazenil) radioligand binding assay (Supplementary Material for detailed methods). The concentration of compound required to inhibit specific binding by 50% (IC_50_) was determined and this was converted to a Ki value using the method of Cheng and Prusoff with the Kd values [^3^H]Ro15–1788 at each of the four different GABA_A_R subtypes.

#### 2.2.2. In vitro efficacy

The effects of the compounds on human recombinant α1β3γ2L, α2β3γ2L, α3β3γ2L and α5β3γ2L GABA_A_Rs expressed in HEK293 cells were evaluated using whole cell automated patch clamp electrophysiology on the SyncroPatch 384PE (Nanion, Germany) at Scottish Biomedical Drug Discovery (SBDD, now a part of Sygnature Discovery), Glasgow. Please see Supplementary Material for methods.

### 2.3. In vivo target engagement of alogabat and Compound 100

#### 2.3.1. α5-GABA_A_R occupancy

These studies measured the extent to which alogabat and Compound 100 were able to inhibit the *ex vivo* binding of [^3^H]L655,708 to the benzodiazepine binding site of α5-GABA_A_Rs in hippocampal tissue. Male Sprague-Dawley rats weighing 250–300 g on the day of the experiment (Charles-River, UK) were used and all procedures were performed in accordance with the UK Animals Scientific Procedures Act 1986.

The assay used is based on the *ex vivo* binding assay described by Li et al., 2006 (Li et al., 2006). The assay was modified such that in order to increase the specificity of the assay, all rats were co-dosed with AZD7325 (1 mg/kg p.o.) to block the benzodiazepine sites on the α1-, α2- and α3-GABA_A_Rs (AZD7325 has very low affinity for α5-GABA_A_Rs and therefore at the 1 mg/kg p.o. dose used should not block binding to α5-GABA_A_Rs). Independent groups of animals were used to measure the effects of alogabat at different time points (0.5, 1.5, 2.5, 3.5 h). Please see Supplementary material for detailed methods.

#### 2.3.2. Bioanalysis to determine plasma concentrations of compounds

As part of the receptor occupancy studies, trunk blood was also collected into heparin-lithium blood tubes immediately following decapitation. Plasma was collected by centrifugation of the blood sample and stored at −20 °C before prior to the measurement of drug concentrations using mass spectrometry (Waters Xevo TQ-Smicro; Supplementary material).

### 2.4. Characterization of alogabat and Compound 100 in the MAM model

For MAM model experiments, adult male Sprague-Dawley rat offspring (postnatal day <65) from MAM- and saline-treated dams (Envigo, Indianapolis, USA) were used for the electrophysiological recordings (Fig. 1 C). Animals were housed in groups of two or three and in a temperature-controlled room (22 ± 1 °C) under standard housing conditions with free access to food and water with a 12 h light/dark cycle. All procedures were conducted according to the guidelines established by The National Institutes of Health Guide for the Care and Use of Laboratory Animals and were approved by the Institutional Animal Care and Use Committee at the University of Pittsburgh. Only male rats were used due to limited availability of the drug as well as differences in pathophysiology between males and females (Uliana et al., 2025, 2024) and to correspond to our previous studies (Gill et al., 2014, 2011).

#### 2.4.1. MAM treatment

**Fig. 2.**
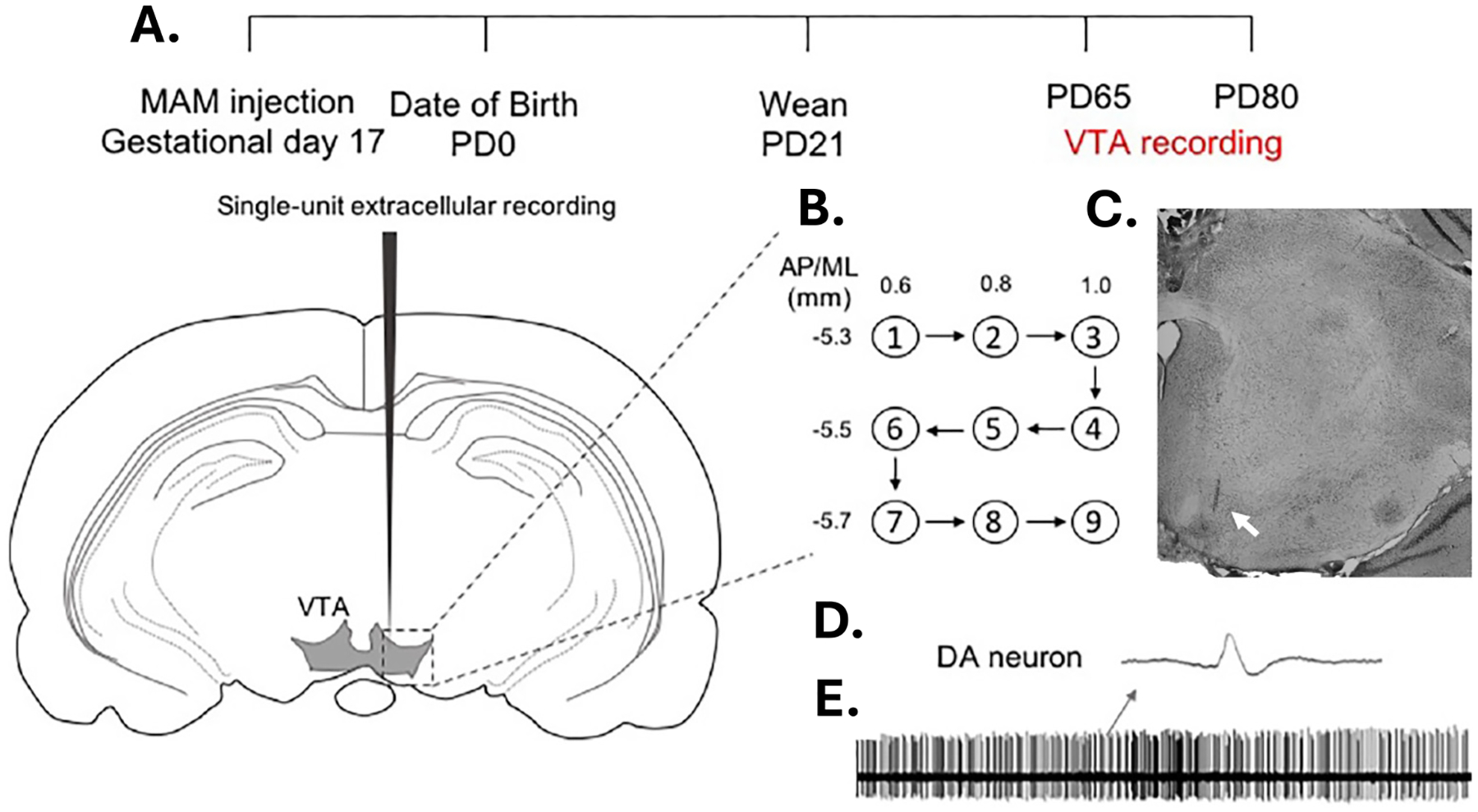
Experimental design to evaluate α5-GABA_A_R PAMs in MAM rats. Electrophysiological recordings of VTA DA activity were evaluated in adult offspring (postnatal day >65) of Saline and MAM-injected pregnant females at gestational day 17 (A). An illustration of the grid pattern for VTA recording with recordings from regions 1–3, 4–6 and 7–9 taking place 0.5–1.5 h, 1.5–2.5 h and 2.5–3.5 h after dosing of compound (and hence the occupancy measurements at 0.5, 1.5 and 2.5 h post-dose) (B), histological placement of electrode within VTA (C), an example of a DA neuron waveform (D), and recording trace (E). MAM: methylazoxymethanol acetate, PD: postnatal day, VTA: ventral tegmental area, DA: dopamine, AP: anteroposterior, ML: mediolateral.

MAM treatment was performed as described in previous studies (Fig. 2A) (Moore et al., 2006). Timed pregnant females were obtained on gestational day 15 and individually housed in ventilated plastic breeding tubs. At gestational day 17, MAM (20 mg/kg, i.p.) or Saline (1 ml/kg, i.p.) was administrated to the dams. On postnatal day 21, males’ offspring were weaned and pair-housed with littermates. Rats were tested during adulthood (postnatal day >65; Fig. 2A). Only two rats from the same dam were used for each treatment/group (drug × veh). For the completion of this study, we used 6 Sal-treated and 6 MAM-treated pregnant rats and 48 offspring.

#### 2.4.2. Drug administration

For the *in vivo* electrophysiology recordings, the 10 mg/kg i.p. of alogabat or 10 mg/kg i.p. Compound 100 were administered 30 min prior to the start of recording in the same vehicle as used for the α5-GABA_A_R occupancy studies (14% propylene glycol/1% Tween80/water; 5 ml/kg). The availability of Compound 100 was insufficient to test the drug under the saline pretreatment conditions and to evaluate receptor occupancy in different time-points (0.5 h only). Accordingly, data with Compound 100 should be considered to be supportive of the more complete data set obtained with alogabat.

#### 2.4.3. Electrophysiological recording

The rats underwent anesthesia with chloral hydrate (400 mg/kg, i.p.) and were subsequently fixed in a stereotaxic frame (Kopf) for electrophysiological recording of DA neurons in the VTA (Supplementary material). DA neuron activity was assessed by counting spontaneously firing DA neurons found during 6–9 vertical passes, spaced by 0.2 μm (Fig. 2B). DA neurons were characterized based on established electrophysiological criteria from previous studies (Fig. 2D) (Grace and Bunney, 1983a; Ungless and Grace, 2012). The activity of each DA neuron was recorded for 3 min (Fig. 2E), and three parameters were measured: (1) population activity, defined as the number of spontaneously active DA neurons recorded per electrode track; (2) firing rate; and (3) the % of action potentials occurring in bursts (burst initiation defined as the presence of two spikes with an interspike interval of 80 ms and termination with 2 spikes >160 ms) (Grace and Bunney, 1983b). VTA data were also analyzed based on electrode location in medial, central, and lateral tracks for population activity, firing rate, and % of spikes in a burst. Additionally, a time analysis was performed in 1-hour block intervals (20 min for each track). Recordings began 30 min after i.p. injection of drugs, and the analysis considered three time-points: 0.5–1.5 h, 1.5–2.5 h, 2.5–3.5 h.

#### 2.4.4. Analysis

The data was represented as the mean ± SEM. The data were assessed for normality (Shapiro-Wilk normality test). One-way ANOVA was used for cells/track analysis in the compound 100 experiment. Two-way ANOVA was used for cells/track data in alogabat experiments, considering treatment (Drugs × Veh) and condition (Sal × MAM) as factors. The firing rate and % spikes in burst were analyzed using the Kruskal-Wallis test because these measures did not pass the normality test. Sidak’s multiple comparison test was used followed by one-way and 2-way ANOVA test. Dunn’s multiple comparison test was used after Kruskal-Wallis. Statistical tests with *p* < 0.05 were considered significant.

## 3. Results

### 3.1. In vitro characterization of alogabat and Compound 100

**Fig. 3.**
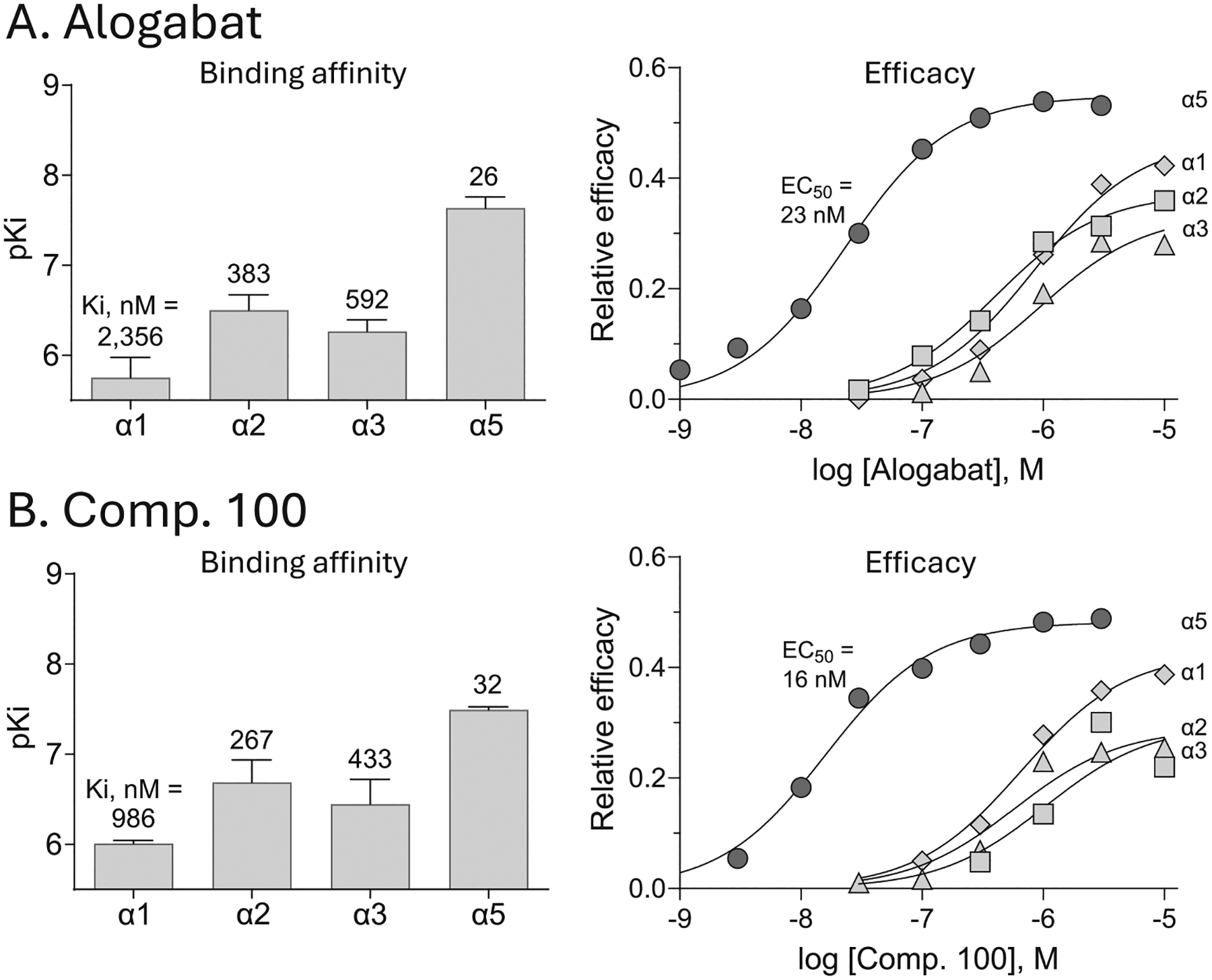
*In vitro* affinity and relative efficacy of (A) Alogabat and (B) Compound 100 at human recombinant GABA_A_ receptors containing β3γ2 subunits and either an α1, α2, α3, or α5 subunit. In the [^3^H]flumazenil radioligand binding assay, both compounds have higher affinity at (*i.e*., binding selectivity for) α5- relative to α1-, α2-and α3-GABA_A_Rs (15–90-fold selectivity for alogabat and 8–31-fold selectivity for Compound 100). Affinities are plotted as pKi with the values above the bars representing the affinity in nM. Data are mean ± SEM (*n* = 3–4). Intrinsic efficacy, measured using whole-cell patch clamp electrophysiology, was defined as the extent to which compound potentiated the current produced by an EC_20_-equivalent of GABA. The extent of this potentiation was then expressed relative to that produced by the non-selective full PAM diazepam (3 μM), which, by definition, has a relative efficacy of 1.0. For clarity, error bars are not shown.

Fig. 3 shows the in vitro binding affinity of PAM efficacy of alogabat and Compound 100 at human recombinant α1-, α2-, α3- and α5-GABA_A_Rs. Both compounds had higher affinity for α5- compared to α1-, α2- and α3-GABA_A_Rs with alogabat and Compound 100 having 15–90-fold and 8–31-fold selectivity for α5-GABA_A_Rs, respectively. In this regard, both compounds are α5-GABA_A_R preferring (binding selectivity <100-fold) rather than α5-GABA_A_R selective (binding selectivity >100-fold).

As regards intrinsic efficacy, both compounds have PAM activity at all four subtypes although the functional affinity of alogabat and Compound 100 (EC_50_ values = 23 and 16 nM) is markedly higher than the EC_50_ values at the other subtypes (EC_50_ values generally in the region of 1 μM). This results, for example, in a concentration of 100 nM (10^−7^ M) having nearly a maximum efficacy at α5-GABA_A_Rs while there is negligible efficacy at the other subtypes.

For alogabat, the binding affinities (*e.g*., Ki at human α5-GABA_A_Rs of 26 nM) and the human GABA_A_R selectivity profiles are very similar to published values of human α5-GABA_A_R Ki of 11 nM which was 17–21-fold higher than other (*i.e*., α1, α2 and α3-containing) GABA_A_R subtypes (Cecere et al., 2025). Moreover, the affinities and selectivity profiles for alogabat at human and rat recombinant GABA_A_Rs were very similar (rat and human α5-GABA_A_R Ki values = 8 and 11 nM, respectively (Cecere et al., 2025)). As regards functional affinity, the EC_50_ value observed in the present study, 23 nM, is comparable to that reported (Cecere et al., 2025) for rat (25 nM) and human (32 nM) α5-GABA_A_Rs. Moreover, our observations of a lower E_max_ and functional affinity (*i.e*., higher EC_50_) for the human α1-, α2- and α3-GABA_A_R subtypes are the same as reported for rat and human GABA_A_Rs (Cecere et al., 2025).

As regards Compound 100, the only data available is that the α5-GABA_A_R Ki value was 8.5 nM and the fold increase in current produced in human α5β3γ2-expressing *Xenopus* oocytes was 1.7-fold. So, at the very least, the data in the patent (WO 2018/104419 A1) showing that Compound 100 is an α5-GABA_A_R PAM are consistent with the current study.

### 3.2. α5-GABA_A_R occupancy by alogabat and Compound 100 in rat brain

**Fig. 4.**
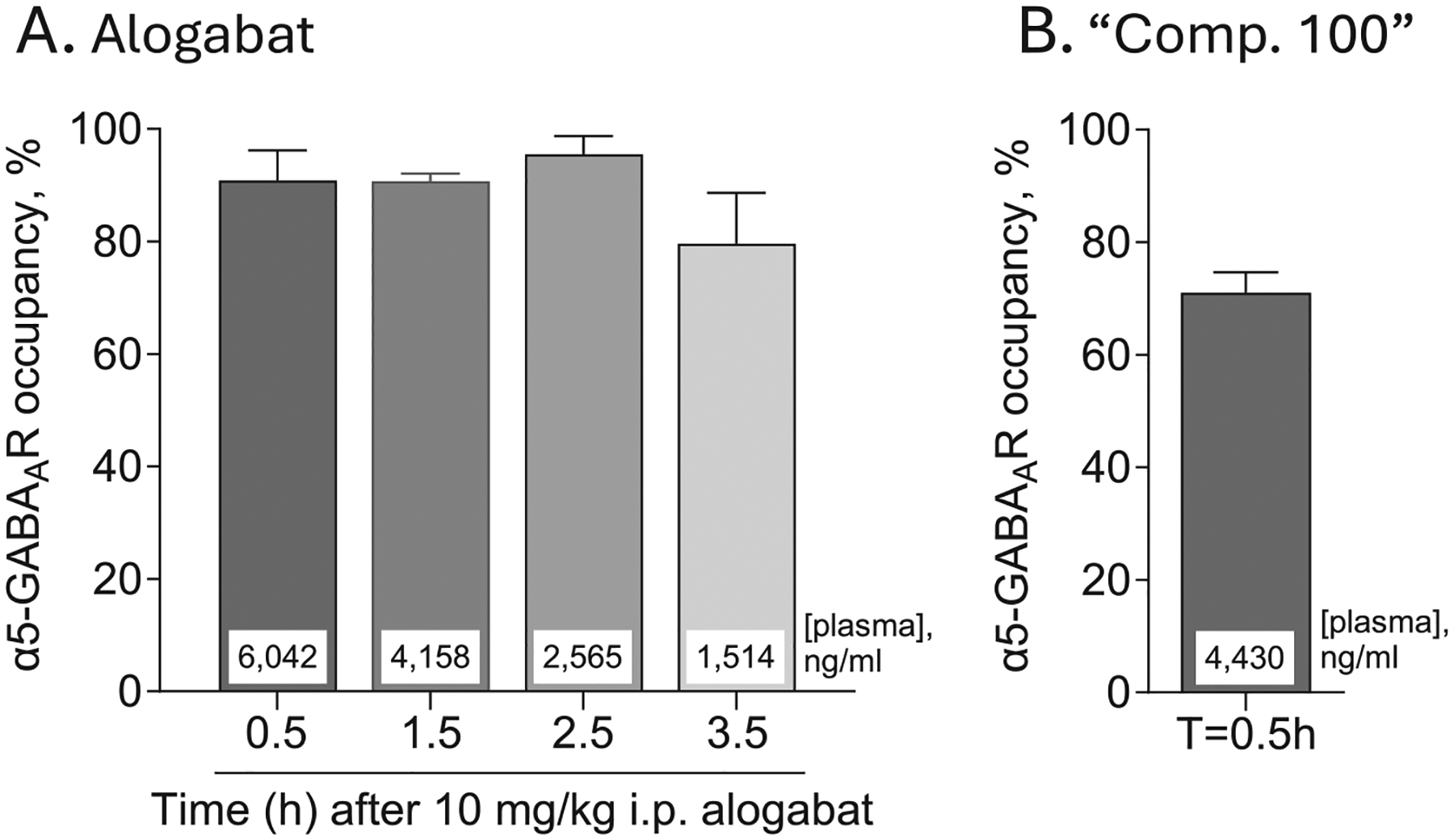
α5-GABA_A_R occupancy measured in male Sprague-Dawley rat hippocampi following doses of 10 mg/kg i.p. alogabat (A) and 10 mg/kg i.p. Compound 100 (B). Plasma was prepared from trunk blood collected from these same animals and drug concentrations in these samples are indicated within each bar. Independent groups of animals were used for each time point after alogabat treatment. Note that there were insufficient amounts of Compound 100 to dose and sample animals at time points other than *t* = 0.5 h. For alogabat, the α5-GABA_A_R occupancy values at 0.5, 1.5, 2.5 and 3.5 h postdose were 91 ± 5, 91 ± 1, 96 ± 3 and 80 ± 9%, respectively whereas 0.5 h after 10 mg/kg i.p. Compound 100, α5-GABA_A_R occupancy was 71 ± 4%. Values shown are mean ± SEM (n = 3–4/group).

In the present study, a dose of 10 mg/kg i.p. of alogabat gave α5-GABA_A_R occupancies of 80–96% at plasma concentrations of 1514 to 6042 ng/ml (Fig. 4A). These values are consistent with the plasma concentration reported to be required to give 50% rat brain α5-GABA_A_R occupancy of a total plasma drug concentration of 669 ng/ml, which in turn resulted in α5-GABA_A_R occupancy (and corresponding plasma drug concentrations) of 57% (1368 ng/ml), c.75% (c.4900 ng/ml) and c.80% (8600 ng/ml) and 5, 15 and 30 mg/kg p.o., respectively (Cecere et al., 2025).

Compound 100 showed an average receptor occupancy of 71% at 0.5 h after 10 mg/kg dose (Fig. 4B), at a plasma concentration of 4430 ng/ml.

### 3.3. Alogabat reverses the increased number of spontaneously active DA neurons

**Fig. 5.**
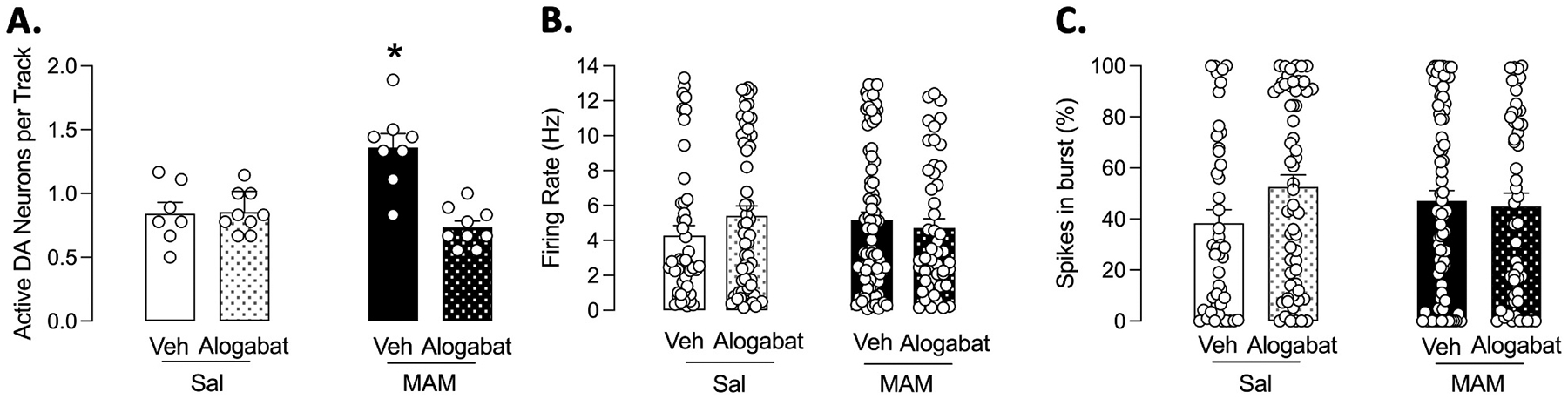
Effects of acute systemic treatment with alogabat (10 mg/kg, i.p.) on VTA DA activity in male MAM rats. Alogabat reversed the increased number of active DA neurons in the VTA of MAM rats (A) with no changes in the DA neurons firing rate (B) and the % of spikes in burst (C). Sal-Veh, *n* = 7 rats and 46 DA neurons; Sal-alogabat, *n* = 9 rats and 60 DA neurons; MAM-Veh, *n* = 8 rats and 83 DA neurons; MAM-alogabat, n = 9 rats and 50 DA neurons. *p < 0.05 Sidak’s multiple comparison *post hoc* and Dunn’s multiple comparison test. MAM: methylazoxymethanol acetate, Veh: vehicle, Sal: saline.

Alogabat treatment did not change the number of DA neurons per track in saline rats (Fig. 5A and Table 1). However, Alogabat significantly reversed the increased number of spontaneously active DA neurons in the VTA of MAM rats (Fig. 5A). Firing rate and % of spikes in burst did not have a normal distribution (All groups, *p* < 0.05; Shapiro-Wilk test) and were analyzed using the Kruskal-Wallis test. No effect was found across all the groups for firing rate (Fig. 5B) and % of spikes in burst (Fig. 5C).

### 3.4. Exploratory analysis based upon neuroanatomical location

Since recording were made in a systematic manner according to the scheme shown in Fig. 2B, it was possible to conduct further, more exploratory analysis in terms of effects of alogabot in the medial, central or lateral VTA, especially given that α5-GABA_A_R occupancy remained high throughout this period (Fig. 4).

**Fig. 6.**
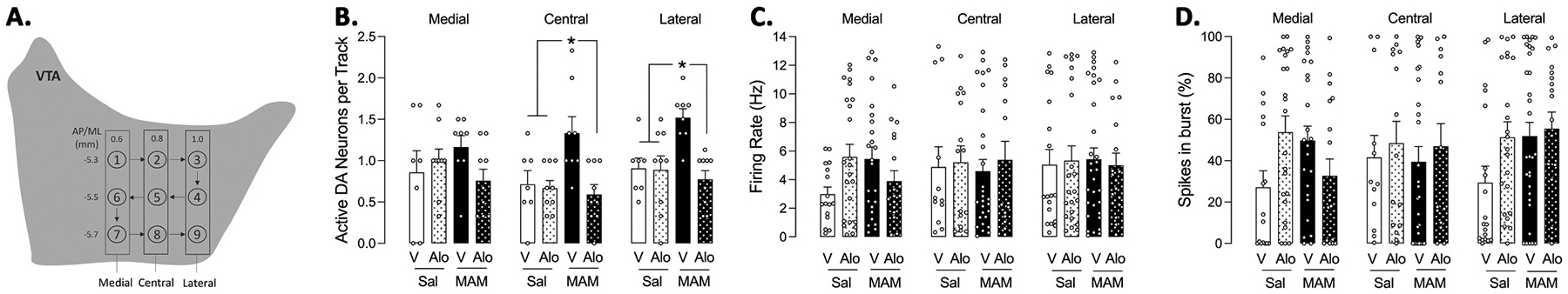
Exploratory analysis of the neuroanatomical effects of alogabat on VTA DA activity in male MAM rats. The VTA data were analyzed based on electrode location within medial, central, and lateral portions of VTA (A). Alogabat decreased the number of active DA neurons in the central and lateral VTA locations in male MAM rats (B). Firing rate and the % of spikes in burst of DA neurons were not altered by Alogabat (C and D) within all portions of VTA. Sal-Veh, n = 7 rats and 12–18 DA neurons; Sal-alogabat, n = 9 rats and 15–23 DA neurons; MAM-Veh, n = 8 rats and 24–31 DA neurons; MAM-alogabat, n = 9 rats and 14–18 DA neurons. *p <0.05 Sidak’s multiple comparison *post hoc*. AP: anteroposterior, ML: mediolateral, VTA: ventral tegmental area, V: Vehicle, Sal: saline, MAM: methylazoxymethanol acetate, Alo: Alogabat.

In the medial-lateral VTA segment analysis, Alogabat reversed the increased number of active DA neurons per track in the central and lateral portions of VTA in MAM rats (Fig. 6B, Table 2). No difference was found in firing rate in the medial, central, and lateral portions of VTA (all portions, *p* > 0.05, Kruskal-Wallis; Fig. 6C). In the medial portion of VTA, an effect was found for % of spikes in bursts (H = 8.53, p < 0.05, Kruskal-Wallis; Fig. 6D) but Dunn’s multiple comparisons test did not indicate a difference between the groups. No effect was observed in the central and lateral portion of VTA for % of spikes in burst (p > 0.05, Kruskal-Wallis).

### 3.5. Exploratory analysis based upon time of analysis

**Fig. 7.**
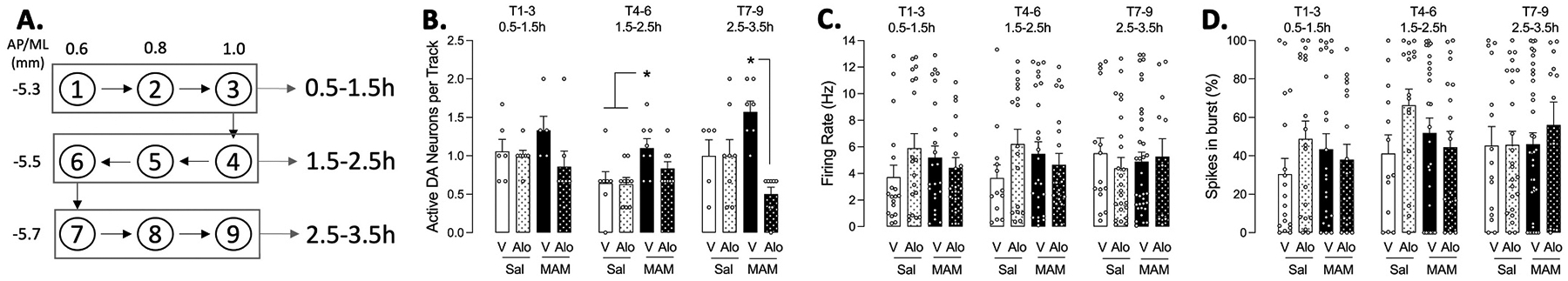
Temporal resolution of the effects of alogabat on VTA DA activity in male MAM rats. The VTA data was analyzed based on 0.5 h–1.5 h, 1.5–2.5 h, and 2.5–3.5 h time blocks corresponding to recording tracks 1–3, 4–6 and 7–9, respectively (A). Alogabat decreased the number of active DA neurons in the VTA during 2.5–3.5 h time-point in male MAM rats (B). Firing rate and the % of spikes in burst of DA neurons were not affected by Alogabat (C and D) at all times. Sal-Veh, *n* = 5–7 rats and 13–18 DA neurons; Sal-alogabat, n = 7–9 rats and 17–24 DA neurons; MAM-Veh, n = 5–8 rats and 23–35 DA neurons; MAM-alogabat, n = 7–8 rats and 12–20 DA neurons. *p < 0.05 Sidak’s multiple comparison *post hoc*. AP: anteroposterior, ML: mediolateral, V: Vehicle, Sal: saline, MAM: methylazoxymethanol acetate, Alo: Alogabat.

Alogabat treatment (10 mg/kg, i.p.) decreased the number of active DA neurons in the VTA in the last time-point only in MAM rat (2.5–3.5 h; Fig. 7B, Table 3). No effect of alogabat treatment was observed during 0.5–1.5 h and 1.5–2.5 h (Fig. 7B, Table 3) in Saline and MAM groups. A significant increase was detected for MAM group treated with vehicle compared to Saline groups during 1.5–2.5 h (Fig. 7B, Table 3). Firing rate and % of spikes in burst did not change across all time and groups (p > 0.05, Kruskal-Wallis; Fig. 7C and D).

### 3.6. Compound 100 reverses the increased number of spontaneously active DA neurons in the VTA of MAM rats

**Fig. 8.**
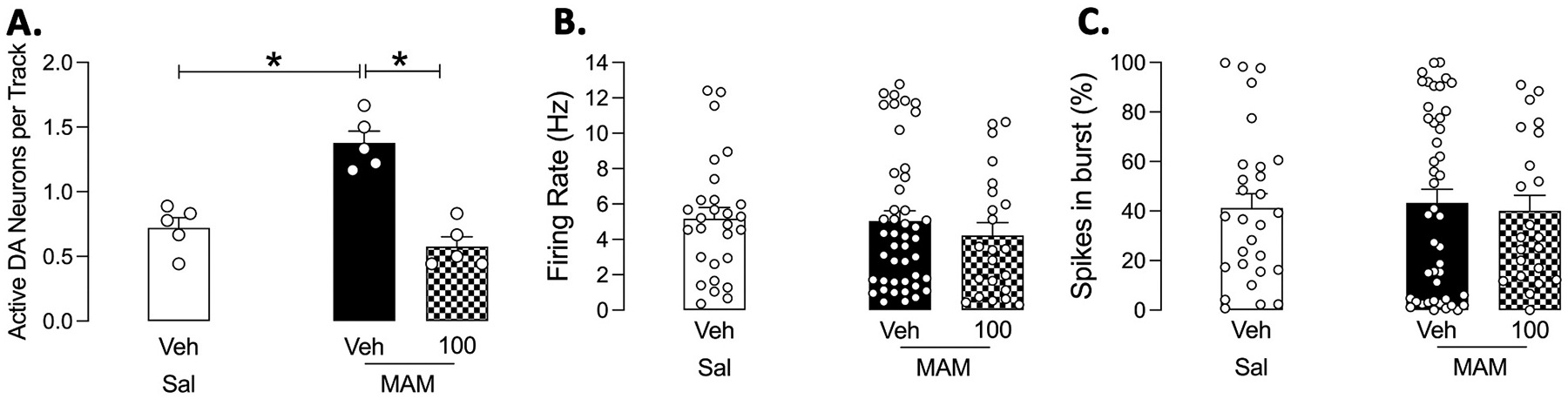
Effects of acute systemic Compound 100 (10 mg/kg) on VTA DA activity in male MAM rats. Compound 100 reversed the increased number of active DA neurons in the VTA in the MAM group (A) but neither the firing rate (B) nor the % of spikes in burst of DA neurons (C) were altered after administration of Compound 100. Sal-Veh, n = 5 rats and 28 DA neurons; MAM-Veh, n = 5 rats and 45 DA neurons; MAM-100, n = 5 rats and 22 DA neurons. *p < 0.05 Sidak’s multiple comparison *post hoc* and Dunn’s multiple comparison test. MAM: methylazoxymethanol acetate, Veh: vehicle, Sal: saline, 100: Compound 100.

Acute treatment with Compound 100 reversed the increased number of spontaneously active DA neurons per track in MAM rats (Fig. 8A; Table 4) to levels that were similar to those observed in the Sal-Veh group. No significant effect was found for firing rate or % of spikes in bursts between the groups (Fig. 8B and C).

The VTA segment analysis indicated that Compound 100 reversed the increased number of spontaneously active DA neurons in the central and lateral portions of VTA (Supplementary Fig. 1). The time analysis demonstrated an effect of treatment in MAM rats during 1.5–2.5 h time-point in cells/track measure but not 0.5–1.5 h and 2.5–3.5 h time-points (Supplementary Fig. 2).

## 4. Discussion

### 4.1. The unmet need for novel therapeutics for the treatment of schizophrenia

The present study demonstrated that two novel α5-GABA_A_R PAMs injected systemically were able to reverse the hyperdopaminergic state in the MAM model. A hyperdopaminergic state in the VTA of rats can be translated to humans as patients with schizophrenia have increased fluorodopa uptake, which is a measure of number of active DA terminals (Lodge and Grace, 2011). It can be also associated with increased amphetamine-induced DA release (Abi-Dargham et al., 2009) reported in the associative striatum of patients with schizophrenia which is correlated with positive symptom severity (Abi-Dargham et al., 2000). Although D2 antagonist antipsychotics are highly effective in decreasing DA activity to treat positive symptoms (Seeman et al., 1975), their side effects along with inefficacy in treating negative and cognitive symptoms contribute to patient non-adherence (Guo et al., 2023; Higashi et al., 2013). Thus, new targets have been studied aiming to find a better and more effective way to treat schizophrenia, such as α5-GABA_A_R PAMs. Different α5-GABA_A_R PAMs were shown effective in restoring the increased DA activity in the VTA (Gill et al., 2011; Perez et al., 2023) which is consistent with our findings showing that alogabat and Compound 100 reversed the increased number of active DA neurons in the VTA of MAM rats.

### 4.2. Neuroanatomical specificity of the effects of alogabat

The VTA is a heterogeneous area that projects to different regions of the striatum, with medial and central portions of VTA projecting to reward-related nucleus accumbens shell area and lateral VTA to associative striatum (Grace, 2016; Ikemoto, 2007). We have demonstrated previously that animal models of schizophrenia, such as the MAM model, have an increased number of spontaneously active DA neurons in the lateral portion of the VTA (Gomes and Grace, 2017; Lodge and Grace, 2012). Thus, we evaluated the number of DA neurons per track, firing rate, and burst firing across medial, central, and lateral VTA segments. Both compounds were able to decrease the number of active DA cells per track in the central and lateral tracks.

One ideal treatment approach for treatment would be to target the pathological site which could remediate multiple symptom classes. The hippocampus is a core area for schizophrenia symptomatology due to hyperexcitability driven by the loss of function and the number of GABAergic interneurons (Lodge and Grace, 2007; Uliana et al., 2023). Thus, in contrast to D2 blocking antipsychotic drugs, by decreasing the hippocampus hyperactivity we would expect to treat the full spectrum of schizophrenia symptomatology (Grace and Uliana, 2023).

### 4.3. Are the effects of alogabat mediated by α5- or other GABA_A_R subtypes?

A key issue that remains is the extent to which the observed attenuation of striatal hyperdopaminergic activity is driven by α5-GABA_A_Rs rather than α1-, α2- and/or α3-GABA_A_Rs. Hence, both alogabat and Compound 100 are α5-preferring rather than α5-selective compounds and while both have higher binding and functional affinity for α5-GABA_A_Rs compared to other subtypes, there nevertheless remains the possibility that some of the attenuation of dopamine hyperactivity is not actually α5-GABA_A_R mediated. Interestingly, it was noted that at α5-GABA_A_R occupancies above 70%, alogabat impaired context and spatial memory (Cecere et al., 2025) but again it is unclear whether this suggests that high levels of α5-GABA_A_R occupancy by a PAM are detrimental or whether this is merely the emergence of side effects driven by effects at the other (α1-, α2- and/or α3 subunit-containing) GABA_A_R subtypes.

A recent study with alogabat demonstrates that this compound positively regulates the inhibitory control of hippocampal pyramidal neurons (Cecere et al., 2025). At doses corresponding to α5-GABAR occupancy of 77–85%, there was increased beta-band activity and decreased theta-band power which is consistent with its facilitation of GABAergic transmission (Cecere et al., 2025). Alogabat reduced repetitive grooming behavior in mouse models of autism without affecting locomotor activity at doses ranging from 60 to 100 mg/kg (receptor occupancy = 45–79%), and it did not produce sedation or ataxia even at 30 mg/kg (receptor occupancy = 88%). However, despite its selective modulation and favorable safety profile, higher doses (10–30 mg/kg) impaired the acquisition of fear and spatial memory, effects that are associated with receptor occupancies exceeding 70–75% (Cecere et al., 2025). In the present study, cognitive effects of alogabat or compound 100 were not evaluated, which represents a limitation. It is possible that these compounds could influence cognitive domains at the tested dose of 10 mg/kg, a dose associated with high levels of α5-GABA_A_R occupancy and cognitive impairment (Cecere et al., 2025). Future studies are required to better investigate the cognitive and behavioral impacts of alogabat and compound 100 at different doses in the context of schizophrenia.

### 4.4. Therapeutic potential of α5-GABA_A_R PAMs in the treatment of schizophrenia

α5-GABA_A_Rs are a promising target for treatment given their high expression in the hippocampus (Fritschy and Panzanelli, 2014; Heldt and Ressler, 2007; Ramos et al., 2004; Serwanski et al., 2006) and the lower α5-GABA_A_R binding present in schizophrenia (Marques et al., 2021) that is associated with negative symptoms (Asai et al., 2008). Furthermore, positive symptom severity correlates with decreased α5-GABA_A_R expression in the limbic hippocampus of unmedicated patients (Marques et al., 2021). Increasing the function of α5-GABA_A_Rs would allow an effective inhibition of excitability in the hippocampus, considering that α5-GABA_A_R mediates tonic inhibitory currents (Bai et al., 2001; Glykys et al., 2008; Glykys and Mody, 2006) and pyramidal neuron activity (Bonin et al., 2007). At the doses used (10 mg/kg i.p.) both compounds achieve substantial occupancy of hippocampal α5-GABA_A_Rs and were able to attenuate the increased DA activity in MAM rats. Previous studies have demonstrated that other α5-GABA_A_R PAMs administered systemically and in the ventral hippocampus decrease the number of spontaneously active DA neurons per track without affecting the firing rate and spikes in burst of the DA neurons in the MAM rats (Gill et al., 2011; Perez et al., 2023) which is similar with our alogabat and compound 100 findings. However, it is described that some GABA_A_R PAMs when administrated directly in the ventral hippocampus can increase or decrease the % of burst firing of DA neurons (Perez et al., 2023). This may suggest that restricted activation of α5-GABA_A_R PAMs within the ventral hippocampus can produce circuit-level alterations that influence areas involved in regulating DA burst firing and stimulus-salience processing, such as the pedunculopontine tegmental nucleus (Juarez and Han, 2016; Mena-Segovia and Bolam, 2017). However, the specific effects on this measure appear to depend on the individual compound rather than being solely attributable to their shared mechanism of action as α5-GABA_A_R PAMs. Additionally, infusion of α5-GABA_A_R PAM in the ventral hippocampus normalized the hyperdopaminergic state in the VTA of stressed rats but not in rats where the α5 overexpression was induced (McCoy et al., 2022). In the MAM model, α5-GABA_A_R overexpression was able to reverse the DA hyperactivity and ventral hippocampus hyperexcitability (Donegan et al., 2019) probably due to the decreased density in the hippocampus (Kiemes et al., 2022) that is not observed in acute stress models (McCoy et al., 2022).

In the MAM model, a non-selective PAM (midazolam) was able attenuate the increased dopaminergic state in MAM rat when it was injected directly in the ventral hippocampus but not systemically (Perez et al., 2023). This may suggest that the effect of a non-selective PAM could be interfering with excitability of other circuits and potentially overcoming the beneficial effect on α5 GABA_A_R in the hippocampus. However, the fact that alogabat and Compound 100 would specifically act on α5-GABA_A_Rs within the hippocampal circuits suggests that they can counteract the increased inputs to the VTA. This functional implication implies that α5-preferring GABA_A_R PAMs may offer a more targeted approach to correcting core circuit dysfunction in schizophrenia, potentially with fewer sedative or tolerance-related side effects compared to nonselective benzodiazepines (Xu and Wong, 2018).

### 4.5. Further considerations

Although the study findings are promising, it also has shortcomings. For example, due to compound availability and in order for comparisons with previous studies, only male MAM rats were used in the present study. A hyperdopaminergic state is present in female MAM rats (Perez et al., 2014; Sonnenschein and Grace, 2020) which is rescued by pharmacological interventions that are effective in male MAM rats (Uliana et al., 2025, 2024). Therefore, one would predict that alogabat (or Compound 100) would also rescue the DA dysfunction observed in females. In addition, as alogabat is α5-GABA_A_R preferring, it would be important to establish whether there is an attenuation of the hyperdopaminergic state at doses that selectively occupy α5-GABA_A_Rs with minimal occupancy at the other subtypes.

In conclusion, this study demonstrated that the α5-GABA_A_R PAMs alogabat reversed the VTA hyperdopaminergic state in the MAM model of schizophrenia by specifically decreasing DA activity in the central and lateral portion of the VTA. These data were confirmed with the use of a second compound, Compound 100. The findings suggest that these α5-GABA_A_R PAMs may have therapeutic potential by targeting hippocampal hyperactivity, which plays a central role in schizophrenia pathophysiology.

## Supplementary Material

1

## Figures and Tables

**Table 1:** Summary of the effects of alogabat on the firing of dopaminergic neurons in the VTA of MAM rats.

Parameter	Maternal treatment	Drug treatment		Statistical analysis
		Veh	Alogabat	
Cell/track	Sal	0.84 ± 0.09	0.85 ± 0.05	Condition, F_1,29_ = 6.81, p < 0.05 Treatment, F_1,29_ = 16.05, p < 0.05 Interaction, F_1,29_ = 17.56, p < 0.05, ANOVA
	MAM	1.36 ± 0.11	0.73 ± 0.05	
Firing rate	Sal	4.3 ± 0.6	5.4 ± 0.6	N.S.
	MAM	5.2 ± 0.5	4.7 ± 0.5	
% spikes in burst	Sal	38.4 ± 5.3	52.5 ± 4.8	N.S.
	MAM	47.1 ± 4.1	44.9 ± 5.2	

**Table 2:** Alogabat: active neurons per track analyzed according to anatomical location within the VTA.

Location	Maternal treatment	Drug treatment		Statistical analysis
		Veh	Alogabat	
Medial	Sal	0.86 ± 0.26	1.0 ± 0.14	N.S.
	MAM	1.16 ± 0.14	0.76 ± 0.14	
Central	Sal	0.71 ± 0.16	0.67 ± 0.09	Condition, p > 0.05 Treatment, F_1,29_ = 7.37, p < 0.05 Interaction, F_1,29_ = 5.7, p < 0.05, ANOVA
	MAM	1.33 ± 0.20	0.59 ± 0.12	
Lateral	Sal	0.90 ± 0.12	0.89 ± 0.17	Condition, p > 0.05 Treatment, F_1,29_ = 8.34, p < 0.05 Interaction, F_1,29_ = 7.58, p < 0.05, ANOVA
	MAM	1.52 ± 0.11	0.77 ± 0.11	

**Table 3:** Active DA neurons per track in the VTA analyzed across time for Alogabat.

Time	Maternal treatment	Drug treatment		Statistical analysis
		Veh	Alogabat	
0.5–1.5 h	Sal	1.06 ± 0.16	1.0 ± 0.07	N.S.
	MAM	1.33 ± 0.18	0.86 ± 0.2	
1.5–2.5 h	Sal	0.64 ± 0.15	0.63 ± 0.09	Condition, F_1,28_ = 8.93, p < 0.05 Treatment, p > 0.05 Interaction, *p* > 0.05, ANOVA
	MAM	1.1 ± 0.12	0.83 ± 0.09	
2.5–3.5 h	Sal	1.0 ± 0.21	1.0 ± 0.21	Condition, p > 0.05 Treatment, F_1,28_ = 9.99, p < 0.05 Interaction, F_1,28_ = 10.07, p < 0.05, ANOVA
	MAM	1.57 ± 0.14	0.5 ± 0.09	

**Table 4:** Summary of the effects of Compound 100 on the firing of dopaminergic neurons in the VTA of MAM rats.

Parameter	Maternal treatment	Drug treatment		Statistical analysis
		Veh	Compound 100	
Cell/track	Sal	0.72 ± 0.08	N/A	F_2,12_ = 26.77, p < 0.05, ANOVA
	MAM	1.38 ± 0.09	0.58 ± 0.08	
Firing rate	Sal	5.17 ± 0.63	N/A	N.S.
	MAM	5.02 ± 0.58	4.21 ± 0.74	
% spikes in burst	Sal	41.22 ± 5.8	N/A	N.S.
	MAM	43.28 ± 5.5	40.05 ± 6.32	

## Data Availability

The data supporting the findings of this study are available upon request.

## Appendix A. Supplementary data

Supplementary data to this article can be found online at https://doi.org/10.1016/j.schres.2026.02.010.
