# Supplementary material for: Novel positive allosteric modulators of alpha 5 subunit-containing GABAA receptors (α5-GABAARs) reverse the hyperdopaminergic state in a neurodevelopmental model of schizophrenia
Source: Schizophr Res. Author manuscript; Available in PMC 2026 Jun 18. (PMC13277557; doi:10.1016/j.schres.2026.02.010)
Supplement: 1 [file NIHMS2178266-supplement-1.docx]

Supplementary Material

**Positive allosteric modulators of alpha 5 subunit-containing GABA_A_ receptors (α5-GABA_A_Rs) reverse the hyperdopaminergic state in a neurodevelopmental model of schizophrenia.**

1. **Material and Methods.**

**1.1. In vitro characterisation of Alogabat and Compound 100**

***1.1.1. Radioligand binding***

In brief, cell membranes were prepared and then 20-50 µg protein/well were incubated with 4 nM [^3^H]Ro15-1788 (83 Ci/mmol, PerkinElmer) in the presence of increasing concentrations of test compound. All incubations were performed for 1 h at room temperature in a total assay volume was 0.5 mL and nonspecific binding being measured in the presence of 3 µM TP003 (Dias et al., 2005). Incubations were terminated by filtration and washing with ice cold Tris-HCl buffer (50 mM, pH = 7.4) over Whatman GF/B filters and the radioactivity of the filters was measured using a Tricarb 2900 liquid scintillation analyser.

- - 1. ***In vitro efficacy***

The SyncroPatch stacked addition protocol was used, in which GABA was rapidly applied and then washed off from the cell. All experiments were carried out at room temperature using a standard whole cell patch clamp procedure. A stable baseline current was established and then the effects of compounds were evaluated in the presence of a submaximal GABA concentration, giving typically 10-20% activation (GABA EC_10-20_) of the response elicited by a saturating GABA concentration. Following a further wash step, a saturating concentration of GABA (10 mM) was then applied, allowing the accurate evaluation for each cell of the percentage baseline activation elicited by the submaximal GABA applied. The relative efficacy to diazepam was calculated for each recording by dividing the compound % efficacy to the diazepam % efficacy.

- 1. **In vivo target engagement of Alogabat and Compound 100**
     1. ***α5-GABA_A_R occupancy***

Rats were dosed with AZD7325 (3 mg/kg p.o. in a vehicle of 14% propylene glycol/1% Tween80/water; 5 mL/kg) and either vehicle, 10 mg/kg i.p. alogabat or 10 mg/kg i.p. Compound 100 (n=3-4/group) with non-specific binding being defined using 10 mg/kg p.o. TPA023 (0.5% methylcellulose suspension, 5 mL/kg; n=3). Animals were culled by stunning and decapitation at times (0.5, 1.5, 2.5 and 3.5 h) pre-determined to correspond to the times of *in vivo* electrophysiology data collection in the MAM animals. At these times, trunk blood and brain samples (hippocampi) were collected. The dissected hippocampi from both hemispheres were weighed promptly and processed further promptly for receptor occupancy evaluation. A minimum of 50 mg per rat was rapidly homogenized in 34x volumes of ice-cold assay buffer (10 mM potassium phosphate buffer, pH 7.0) and 1350 μL aliquots of this homogenate were added to 150 μL of 5 nM [^3^H]L655,708 in assay buffer, on ice (final [^3^H]L655,708 concentration of 0.5 nM). All samples were assayed in triplicate and vortexed immediately and incubated for 20 s on ice and incubations were terminated by filtration through Whatman GF/B filters and washing with 10 mL of ice-cold 50 mM Tris buffer (pH 7.4). The radioactivity retained on the filters was determined by liquid scintillation counting using a Packard TriCarb2900 Liquid Scintillation Analyzer. The α5-GABA_A_R occupancy of the test compound was calculated as the percentage by which the specific binding of [^3^H] L655,708 was inhibited by drug relative to vehicle-treated animals.

- - 1. ***Bioanalysis to determine plasma concentrations of compounds***

Analyte standard curve samples and plasma samples were precipitated in MeOH containing internal standard (carbamazepine). Once centrifuged, the supernatant was diluted and subjected to HybridSPE filtration to remove lipids to minimise matrix-associated ion suppression during MS analysis, followed by vacuum evaporation. The dried samples were then re-suspended in 50% MeCN and analysed via LC/MS-MS. Analyte peak areas were normalized to the internal standard and concentrations interpolated from the matrix matched standard curve.

- 1. **Characterisation of Alogabat and Compound 100 in the MAM model**
     1. ***Electrophysiological recording***

After the rat was fixed in the stereotaxic the body temperature was maintained at of 37°C with a temperature-controlled heating pad (Fintronics). Anesthesia was supplemented through periodic i.p. injections of chloral hydrate, adjusting the dosage as needed to suppress the hindlimb compression withdrawal reflex. *In vivo* extracellular recordings were performed using electrodes constructed from Omegadot 2.0 mm glass tubing and pulled using a vertical electrode puller (Narishige P-5, Japan). Electrodes were filled with a solution containing 2 M NaCl and 2% Sky Blue dye. The electrodes were lowered into the ventral tegmental area (VTA) at the following coordinates: AP -5.3 mm from bregma, ML +0.6 mm from midline, and 6.5 to 9.0 mm ventral from the brain surface. The ventral positioning was reached using a hydraulic microdrive system (Kopf). Neural activity was assessed using an open filter setting (low pass=50 Hz; high pass=16 kHz).

- - 1. ***Histology***

Upon the termination of electrophysiological recording, the electrode location was marked with an electrophoretic ejection of Chicago sky blue dye using -20mA constant negative current for 20 min (Figure 2C). Euthanasia was induced with a lethal dose of chloral hydrate with an additional 400 mg/kg dose by the end of electrode site marking. The rats were decapitated and the brains were removed and stored in an 8% paraformaldehyde solution for 48 h for tissue fixing. The brains were transferred to a 25% sucrose solution for cryoprotection. The brains were sliced coronally (60 mm) using a cryostat (Leica Frigocut 280), mounted on gelatin-chormalum-coated slides, and stained with a combination of neutral red and cresyl violet.

1. ***Results***
   1. **Compound 100 reverses the increased number of spontaneously active DA neurons in the VTA of MAM rats.**

******

**Supplementary Figure 1. Analysis of the neuroanatomical effects of Compound 100 on VTA DA activity in male MAM rats. T**he VTA data were analyzed based on electrode location within medial, central, and lateral portions of VTA. Compound 100 decreased the number of active DA neurons in the central and lateral VTA locations in male MAM rats **(A)**. Firing rate and the % of spikes in burst of DA neurons were not altered by Compound 100 **(B and C)** within all portions of VTA. *p<0.05 Sidak’s multiple comparison post hoc. VTA: ventral tegmental area, V: Vehicle, Sal: saline, MAM: methylazoxymethanol acetate, 100: Compound 100.

******

**Supplementary Figure 2. Temporal resolution of the effects of Compound 100 on VTA DA activity in male MAM rats.** The VTA data was analyzed based on 0.5h-1.5h, 1.5-2.5h, and 2.5-3.5h time blocks corresponding to recording tracks 1-3, 4-6 and 7-9, respectively.Compound 100 decreased the number of active DA neurons in the VTA during 1.5-2.5h time-point in male MAM rats **(A)**. Firing rate and the % of spikes in burst of DA neurons were not affected by Compound 100 **(B and C)** at all times. *p<0.05 Sidak’s multiple comparison post hoc. V: Vehicle, Sal: saline, MAM: methylazoxymethanol acetate, 100: Compound 100.
